# MDF-Det: Motion-Aware Decoupling and Scene Filtering for Wide Area Small Moving Target Detection

**DOI:** 10.3390/s26185820

**Published:** 2026-09-14

**Authors:** Kangqiushi Li, Xiaoran Zhang, Zheng Zhang, Huaxin Xiao, Yu Liu

**Affiliations:** College of Systems Engineering, National University of Defense Technology, Changsha 410073, China; likangqiushi20@nudt.edu.cn (K.L.); zhangxiaoran@nudt.edu.cn (X.Z.); xiaohuaxin@nudt.edu.cn (H.X.); jaspnyuliu@nudt.edu.cn (Y.L.)

**Keywords:** wide area motion imagery, small moving target detection, motion-appearance fusion, target decoupling, scene-prior filtering

## Abstract

**Highlights:**

**What are the main findings?**

- MDF-Det establishes a unified coarse-to-fine framework that jointly addresses weak-target preservation, dense-target decoupling, and scene-induced false-alarm suppression in wide-area motion imagery.
- The spatial attention-guided decoupling and learned scene-prior filtering mechanisms separate merged target responses and suppress contextually implausible detections, enabling MDF-Det to achieve an average *F*_1_ score of 0.878 across six WPAFB AOIs.

**What are the implications of the main findings?**

- The results demonstrate that reliable WAMI detection requires coordinated control of candidate recall, dense-target separation, and scene-level contextual filtering rather than relying solely on local motion cues.
- The proposed modular framework provides an interpretable and extensible solution for detecting tiny and densely distributed moving targets under complex wide-area aerial imaging conditions.

**Abstract:**

Object detection in Wide Area Motion Imagery (WAMI) is crucial for large-scale intelligent surveillance and monitoring systems. However, detecting extremely small moving targets in low-frame-rate grayscale WAMI remains highly challenging. In particular, three key problems limit the performance of existing methods: weak motion responses caused by low target contrast can lead to missed detections; densely distributed targets often produce merged responses that are difficult to separate; and registration artifacts, parallax, and dynamic background clutter can generate numerous false alarms. To address these problems, we propose MDF-Det, a coarse-to-fine spatiotemporal framework for WAMI small moving target detection. The framework consists of three complementary components. First, a Motion and Appearance Feature Fusion (MAFF) strategy integrates dense optical-flow-derived motion saliency with grayscale frame differencing to enhance weak target responses and improve candidate preservation. Second, a Spatial Attention-Guided Target Decoupling (SA-TD) module employs fine-grained heatmap decoding and step-threshold degradation to separate merged responses in densely populated scenes. Finally, a Scene-Prior Guided Filtering (SPGF) mechanism learns complementary vehicle-accessibility and motion-activity priors from scene context to suppress contextually implausible false alarms caused by complex background interference. Extensive experiments on six evaluation AOIs of the WPAFB 2009 dataset demonstrate that MDF-Det achieves an average $F_{1}$ score of 0.878, corresponding to a relative improvement of 5.5% over the strongest baseline.

## 1. Introduction

Wide-Area Motion Imagery (WAMI) is an advanced aerial imaging technology that enables persistent coverage of large geographical areas, often spanning entire cities or tens of square kilometers [1]. The detection and tracking of moving objects such as vehicles and pedestrians in WAMI constitute important tasks for a range of emerging applications, including urban monitoring, critical infrastructure protection, disaster response, border surveillance, and defense [2,3,4]. Recent studies have also explored neuromorphic event cameras for WAMI, demonstrating their potential for effective aerial sensing under low-frame-rate and challenging illumination conditions [5]. With continued advancements in sensor and platform technology, WAMI data collection has become increasingly accessible, driving a growing demand for object detection and tracking algorithms to support effective wide-area surveillance.

**Figure 1:**
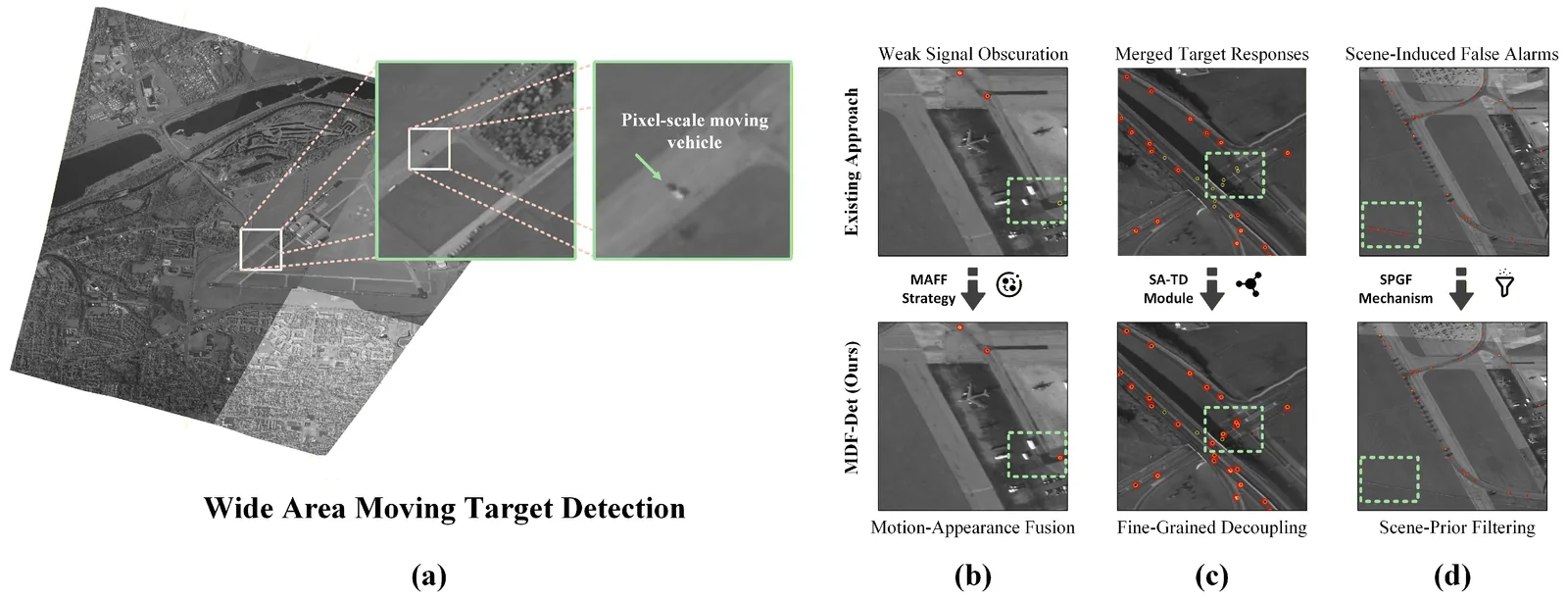
Conceptual illustration of MDF-Det for wide-area moving target detection. (**a**) Full-scale WAMI scene with zoomed-in pixel-scale vehicles. (**b**–**d**) Comparison with conventional methods under weak signal obscuration, merged target responses, and scene-induced false alarms. MDF-Det addresses these challenges through motion-appearance fusion, fine-grained decoupling, and scene-prior filtering, respectively. Green dashed boxes mark representative regions, red circles denote predictions, and yellow circles denote ground truths.

However, object detection in WAMI presents unique and formidable challenges when compared to the task of object detection in ordinary videos [1,6,7]. First, the individual frames have extremely high resolutions. For example, the WPAFB dataset [8] contains frames exceeding 315 megapixels, whereas moving vehicles typically occupy only about 50 pixels on average [9], as illustrated in Figure 1a. Second, WAMI data are often acquired at a low frame rate (typically about 1∼2 frames per second). This slow temporal sampling causes fast-moving vehicles to exhibit large interframe displacements that often exceed the target’s own size, which severely undermines conventional motion-based methods that rely on small, smooth motion assumptions [6,10]. Third, these tiny targets are densely distributed across complex, heterogeneous aerial backgrounds, making efficient and accurate localization highly challenging.

To address these challenges, WAMI object detection has evolved from traditional motion-based techniques [4,10] to deep spatiotemporal frameworks [6,11,12]. Yet, despite these advances, the extreme imaging conditions of WAMI still expose several critical limitations in existing approaches, as illustrated in Figure 1. First, the extremely small target size, weak appearance contrast, and complex aerial backgrounds often obscure subtle motion responses, making faint targets difficult to preserve during candidate generation (Figure 1b) [6,10]. Second, in high-density traffic scenes, neighboring vehicles may produce highly overlapping responses, which severely hinders conventional heatmap regression from separating densely packed targets (Figure 1c) [3,4,12]. Third, without scene-level contextual constraints, existing detectors may misclassify registration artifacts, building parallax, swaying vegetation, and other deceptive background responses as genuine moving targets, resulting in numerous contextually implausible false positives (Figure 1d) [3,10].

To deal with these problems, we propose MDF-Det, a coarse-to-fine framework for vehicle detection in WAMI. By integrating temporal motion priors with grayscale appearance-change features, the proposed framework adaptively enhances low-contrast targets and subtle motion signals at the front end. It then exploits spatial attention mechanisms to separate densely clustered targets in the deep feature space, while further incorporating scene-level contextual priors to suppress background artifacts. Together, these components form an effective cascaded detection pipeline that addresses the inherent challenges of wide-area small moving target detection.

The main contributions of this work are summarized as follows:

- We propose MDF-Det, a unified coarse-to-fine framework for small moving target detection in WAMI. The proposed cascaded pipeline sequentially improves candidate quality through motion–appearance fusion, separates densely clustered targets, and suppresses false positives via scene-level filtering.
- We develop a Motion and Appearance Feature Fusion (MAFF) strategy. By integrating temporal motion saliency derived from optical flow with grayscale difference features, MAFF adaptively enhances low-contrast signals, thereby improving the detection capability for faint targets.
- We design a Spatial Attention-Guided Target Decoupling (SA-TD) module. By combining fine-grained heatmap decoding with a step-threshold degradation algorithm, the proposed module effectively separates overlapping targets in highly crowded scenarios.
- We introduce a Scene-Prior Guided Filtering (SPGF) mechanism. By learning complementary vehicle-accessibility and motion-activity priors from trajectory-supervised scene context, SPGF suppresses false positives caused by background clutter in contextually implausible regions.
- Extensive experiments on the WPAFB 2009 dataset demonstrate that MDF-Det achieves the highest $F_{1}$ scores across all six evaluation AOIs, with an average $F_{1}$ score of 0.878, corresponding to a relative improvement of 5.5% over the strongest baseline.

The remainder of this paper is organized as follows. Section 2 reviews the literature on moving object detection in WAMI. Section 3 presents the proposed MDF-Det framework and its core components. Section 4 describes the dataset, evaluation metrics, baseline methods, implementation details, and network training settings. Section 5 presents the experimental results. Finally, Section 6 discusses the main findings and limitations, and Section 7 concludes the paper.

## 2. Related Work

The primary objective of object detection in WAMI is to efficiently localize and track high-value dynamic targets, such as moving vehicles, across gigapixel-scale aerial backgrounds. Based on methodological evolution, existing detection approaches can be broadly categorized into three distinct classes: traditional techniques relying on temporal motion cues, two-stage spatiotemporal architectures, and end-to-end single-stage networks driven by heatmap regression.

### 2.1. Traditional Methods Utilizing Temporal Motion Cues

Early research in WAMI primarily relies on temporal motion cues to compensate for the scarcity of spatial appearance details. Background subtraction and multi-frame differencing constitute the foundational techniques for extracting motion pixels from quasi-static backgrounds. Although initial approaches frequently employ two- or three-frame differencing to capture dynamic responses, they suffer from the aperture problem for slow-moving targets and ghosting artifacts induced by the displacement of objects [12]. To adapt to gradual environmental variations, subsequent research introduces more statistical techniques for background modeling—such as adaptive Gaussian Mixture Models [13] and median or mean filtering algorithms [4,14]—to separate moving vehicles from static scenes.

Despite the computational efficiency of these methods, they remain highly susceptible to errors in image registration. Specifically, parallax-induced artifacts frequently generate false positives within these traditional frameworks. Furthermore, they inherently struggle to detect slow-moving targets and objects with low visual contrast against the background.

### 2.2. Two-Stage Architectures with Spatiotemporal Context

To balance the extensive background search space against computational constraints, two-stage coarse-to-fine detection architectures emerge as a pivotal strategy in this domain. Early hybrid frameworks typically employ traditional techniques, such as background subtraction, to generate candidate regions for initial coarse screening. Subsequently, they cascade lightweight Convolutional Neural Networks (CNNs) as backend classifiers to filter out false positives and separate tightly clustered targets [15]. Although these pipelines prioritize efficiency, the overall performance remains highly susceptible to the quality of the initial physical preprocessing.

To circumvent these limitations, subsequent research transitions the two-stage approach toward fully deep convolutional architectures. Pioneering this shift, ClusterNet [11] introduces an anchor-free, fully deep hierarchical framework. In this architecture, the first stage extracts large-scale motion clusters, whereas the second stage performs refined localization via heatmap regression. This design improves detection sensitivity and represents an early deep-learning approach in WAMI for detecting tiny vehicles.

Although two-stage architectures effectively balance search efficiency and detection accuracy, they remain heavily dependent on the quality of proposals generated in the first stage. If a missed detection occurs during this initial phase, the downstream deep networks lack the inherent capacity to recover the target, which ultimately leads to irrecoverable target loss.

### 2.3. Heatmap-Based Single-Stage Regression Networks

Inspired by anchor-free methods such as CenterNet [16], regression based on heatmaps gradually emerges as a leading approach in the field of WAMI. By representing targets as two-dimensional Gaussian keypoints and directly regressing the center positions, these methods effectively bypass the complex design and matching heuristics associated with traditional anchor boxes. Representative networks, such as HM-Net [6] and HMRN [17], extend this concept by incorporating the image and the predicted heatmap of the previous frame as temporal feedback priors alongside the input of the current frame. This architecture enables the joint detection and prediction of target displacement in a single forward pass, thereby efficiently facilitating the tracking of multiple targets.

To capture complex long-range spatiotemporal dynamics, Negin et al. [12] introduce a self-attention mechanism that leverages VQ-VAE to extract unsupervised, discrete spatiotemporal embeddings. These latent embeddings are subsequently integrated into an encoder-decoder architecture based on the Transformer to generate refined heatmaps of keypoints. Furthermore, by incorporating the regularization of shape priors into the loss function, the framework encourages the network to produce responses that align with the physical morphology of small targets.

However, lacking decoupling mechanisms for complex dense scenarios, these methods frequently suffer from overlapping responses in heatmaps when targets are clustered, which leads to a significant degradation in the accuracy of localization.

Limitations and Motivation: In summary, although existing approaches have advanced vehicle detection in WAMI, they still struggle to jointly preserve weak motion responses and separate densely clustered targets during subsequent refinement. Motivated by these limitations, we reformulate the conventional coarse-to-fine detection pipeline and propose MDF-Det. By enhancing faint motion signals through motion priors, separating overlapping targets via fine-grained heatmap decoding, and suppressing contextually implausible false positives using scene-level contextual constraints, MDF-Det provides a unified framework for wide-area moving target detection.

## 3. Proposed Method

### 3.1. Overview

**Figure 2:**
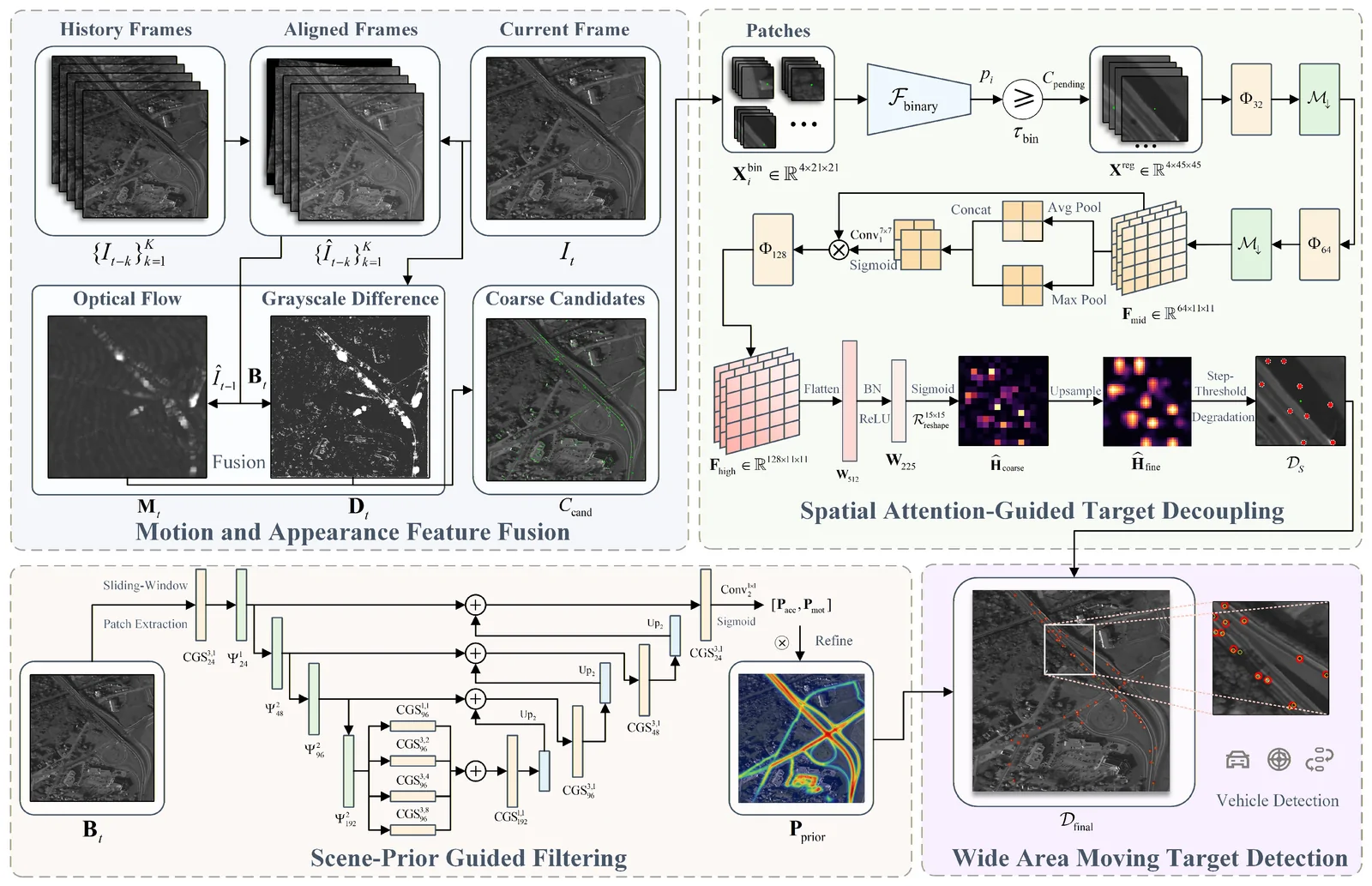
Overview of the proposed MDF-Det framework. The pipeline consists of three core components: MAFF: fuses dense optical-flow motion cues with grayscale differencing to enhance weak target responses and generate coarse candidates; SA-TD: combines spatial attention, fine-grained heatmap decoding, and step-threshold degradation to separate densely clustered targets; and SPGF: learns complementary vehicle-accessibility and motion-activity priors from scene context to suppress contextually implausible false alarms.

As illustrated in Figure 2, MDF-Det adopts a coarse-to-fine architecture for wide-area moving target detection. The pipeline begins with the Motion and Appearance Feature Fusion (MAFF) strategy, which incorporates temporal motion priors to enhance weak target responses and generate coarse candidates. These candidates are subsequently processed by the Spatial Attention-Guided Target Decoupling (SA-TD) module, where fine-grained heatmap decoding separates merged target responses in densely populated scenes. Finally, the Scene-Prior Guided Filtering (SPGF) mechanism exploits learned scene-level contextual priors to suppress false responses caused by parallax and dynamic background clutter, yielding the final detection results.

### 3.2. Motion and Appearance Feature Fusion Strategy

In WAMI, traditional background subtraction algorithms predominantly rely on pixel-level grayscale differences. Consequently, when dealing with low-contrast or slow-moving targets, rigid difference thresholds may lead to missed detections [18]. To improve candidate region extraction, we propose the MAFF strategy, which combines two complementary cues. Specifically, dense optical flow provides explicit displacement-based motion information by estimating inter-frame pixel motion, whereas grayscale differencing captures photometric appearance changes through local intensity variations between the current frame and the temporally aligned reference background. MAFF integrates these motion and appearance-change cues to enhance weak target responses while preserving the efficiency of the conventional grayscale difference framework.

#### 3.2.1. Spatial Registration and Photometric Compensation

Given a current frame $I_{t}$ and a sequence of historical frames ${\left\{I_{t-k}\right\}}_{k=1}^{K}$, our initial objective is to eliminate the global background displacement induced by the ego-motion of the aerial platform. To achieve this, we first employ the ORB feature extractor [19] to compute image correspondences and estimate the global homography matrix $T_{k}\in R^{3\times 3}$, which maps each historical frame to the current frame. Utilizing this matrix, we execute a perspective transformation [20] to project the historical frames into the unified spatial coordinate system of $I_{t}$. This spatial registration process yields the aligned template set, denoted as ${\left\{{\tilde{I}}_{t-k}\right\}}_{k=1}^{K}$:

(1) $${\tilde{I}}_{t-k}\left(x\right)=I_{t-k}\left(W(x;T_{k}^{-1})\right)$$

where $x={\left[x,y\right]}^{T}$ denotes the two-dimensional pixel coordinates in the current frame, and W(·) represents the spatial warping and bilinear resampling operator parameterized by the inverse homography matrix. During the registration process, out-of-bounds regions and void areas lacking pixel values—induced by the perspective transformation—are systematically extracted and morphologically dilated to construct a global invalid mask, $\Omega_{\mathrm{invalid}}$, for subsequent processing.

Despite achieving precise spatial alignment, dynamic factors such as cloud occlusion and viewpoint variations frequently introduce significant photometric inconsistencies. Consequently, prior to constructing the reference background, we apply a large-scale Gaussian low-pass filter, denoted as $G_{\mathrm{illum}}$, to perform adaptive photometric compensation on the aligned templates [15]. This operation effectively isolates the low-frequency illumination drift components:

(2) $${\hat{I}}_{t-k}=\mathrm{Clip}\left({\tilde{I}}_{t-k}+G_{\mathrm{illum}}\left(I_{t}-{\tilde{I}}_{t-k}\right),\;0,255\right)$$

where Clip(·,0,255) is a clipping function that bounds the pixel values within the standard grayscale interval of [0,255]; and ${\hat{I}}_{t-k}$ represents the resulting photometrically aligned template.

Utilizing the photometrically aligned template set ${\left\{{\hat{I}}_{t-k}\right\}}_{k=1}^{K}$, we construct a local reference background, $B_{t}$, via temporal median filtering Median(·). Subsequently, we compute the initial absolute grayscale difference map $D_{t}$:

(3) $$B_{t}\left(x,y\right)={\mathrm{Median}}_{1\leq k\leq K}\left\{{\hat{I}}_{t-k}\left(x,y\right)\right\},\;D_{t}=|I_{t}-B_{t}|$$

#### 3.2.2. Enhanced Detection and Candidate Generation

To overcome the inherent limitations of traditional grayscale differencing—which frequently fails to detect low-contrast, slow-moving targets—we adopt a dense optical flow algorithm based on local polynomial expansion [21,22]. This approach explicitly captures subtle motion information, thereby enhancing weak motion responses during candidate generation. Specifically, we extract the pixel-level motion field $d={\left(u,v\right)}^{T}$ between the current frame $I_{t}$ and the preceding frame ${\hat{I}}_{t-1}$ following spatial registration and photometric compensation. For a given spatial coordinate $x={\left[x,y\right]}^{T}$, the algorithm first approximates the image intensity signal within its local neighborhood as a quadratic polynomial surface:

(4) $$I_{t}\left(x\right)\approx x^{T}A_{t}x+b_{t}^{T}x+c_{t}$$

where $A_{t}$ denotes a symmetric matrix encoding the local spatial second-order derivatives; $b_{t}$ is a column vector representing the first-order spatial gradients; and $c_{t}$ is a scalar corresponding to the locally smoothed central grayscale intensity.

Assuming the target satisfies the brightness constancy constraint over an infinitesimal time interval—formally expressed as $I_{t}\left(x\right)={\hat{I}}_{t-1}\left(x-d\right)$—equating the polynomial coefficients of adjacent frames yields the ideal theoretical constraint: $A_{t}d=-\frac{1}{2}\left(b_{t}-{\hat{b}}_{t-1}\right)$. However, to ensure effective motion estimation in the presence of image noise, we formulate a weighted least squares optimization objective with respect to the displacement vector d over a local spatial neighborhood Q:

(5) $$d^{*}\left(x\right)=\arg\min_{d}\sum_{\Delta x\in Q}w\left(\Delta x\right){∥A\left(x+\Delta x\right)d-\Delta b\left(x+\Delta x\right)∥}^{2}$$

where $A\left(\cdot \right)=\frac{1}{2}\left(A_{t}\left(\cdot \right)+{\hat{A}}_{t-1}\left(\cdot \right)\right)$ denotes the arithmetic mean of the second-order coefficient matrices across the adjacent frames; $\Delta b=-\frac{1}{2}\left(b_{t}-{\hat{b}}_{t-1}\right)$ represents the scaled difference vector of the first-order gradients; Δx indicates the spatial coordinate offset within the local neighborhood Q; and w(·) is a two-dimensional Gaussian weighting function that governs the neighborhood contributions.

Following the estimation of the optical flow field, we derive a normalized two-dimensional temporal motion saliency map, $M_{t}\in {\left[0,1\right]}^{W\times H}$, by computing the pixel-wise motion magnitude and subsequently applying a small-kernel Gaussian smoothing filter, $G_{\mathrm{flow}}$:

(6) $$M_{t}=N\left(G_{\mathrm{flow}}\left(\sqrt{u^{2}+v^{2}}\right)\right)$$

where *W* and *H* denote the spatial dimensions (width and height) of the input image, respectively; and N(·) represents a linear normalization operator that scales the local motion magnitude into the interval [0,1].

Subsequently, we introduce an independent additive motion offset term to explicitly integrate the motion saliency as a physical prior, thereby yielding the enhanced difference map $D_{t}^{\prime }$:

(7) $$D_{t}^{\prime }=\left\{\begin{array}{cc} D_{t}, & \mathrm{if}\;\left(x,y\right)\in \Omega_{\mathrm{invalid}}\\ D_{t}+\alpha \left(M_{t}-T_{m}\right), & \mathrm{otherwise} \end{array}\right.$$

where $M_{t}$ denotes the estimated motion magnitude; $T_{m}$ represents a static noise threshold; and α is a scaling factor that modulates the motion offset. This additive formulation selectively amplifies the signal in dynamic regions ($M_{t}>T_{m}$) while penalizing static background responses.

Subsequently, we apply a binarization threshold, $\tau_{\mathrm{diff}}$, to extract the potential target regions:

(8) $$B_{\mathrm{cand}}\left(x\right)=I\left(D_{t}^{\prime }\left(x\right)\geq \tau_{\mathrm{diff}}\right)$$

where $B_{\mathrm{cand}}$ denotes the resulting binary candidate mask, wherein pixels with a value of 1 designate potential moving targets; I(·) is the standard indicator function.

**Figure 3:**
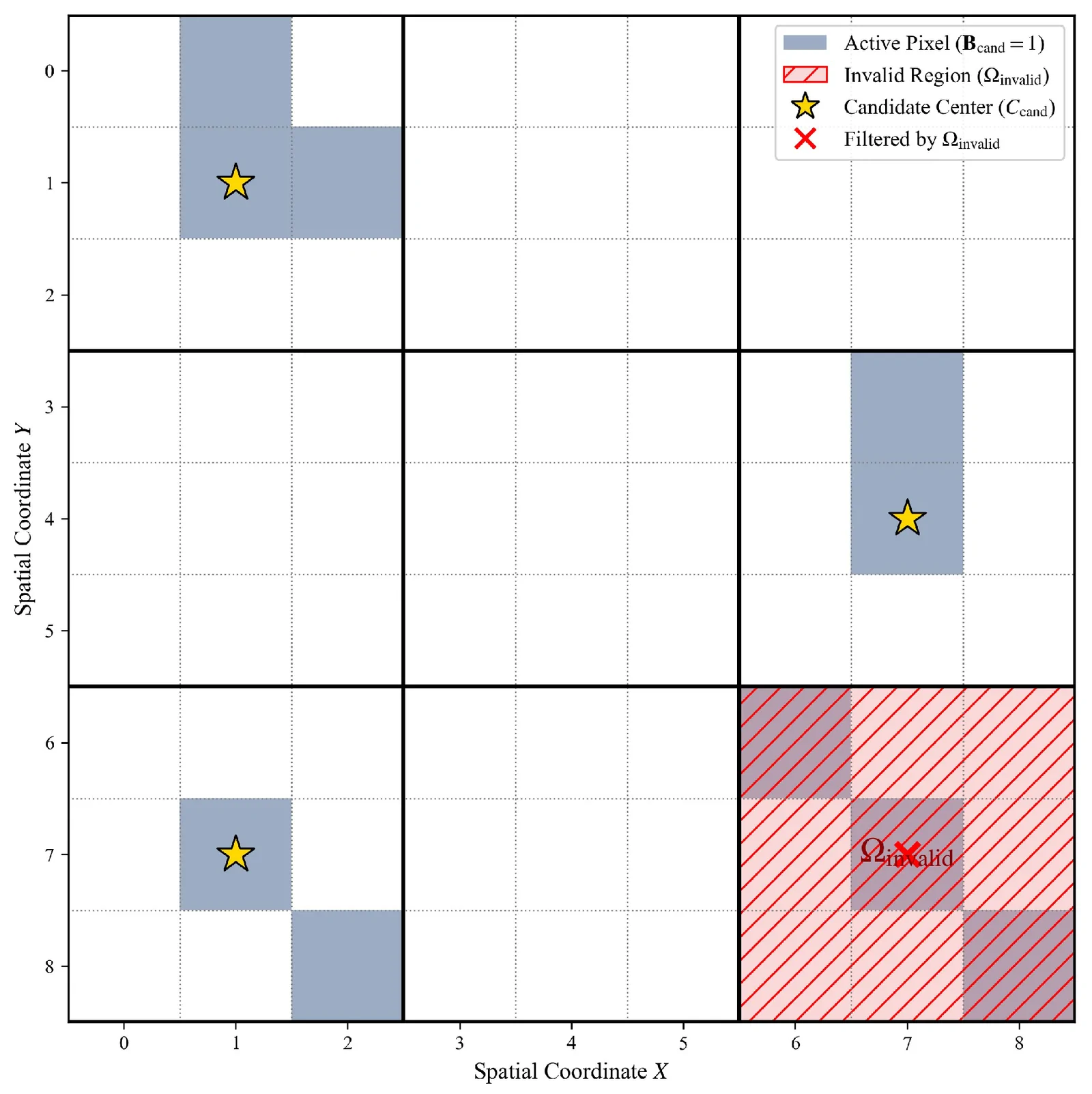
Illustration of the center-anchored candidate extraction. Locally aggregated active pixels (blue) in $B_{\mathrm{cand}}$ are downsampled into unified candidate centers (yellow stars) via grid-based max pooling, while redundant responses within the global invalid boundary $\Omega_{\mathrm{invalid}}$ are explicitly suppressed.

Following the application of morphological denoising to $B_{\mathrm{cand}}$, we partition the binary mask into 3×3 non-overlapping spatial cells to facilitate lightweight, grid-based non-maximum suppression and eliminate redundant, locally clustered responses [23]. As illustrated in Figure 3, by jointly executing local max pooling and center-anchored mapping, the extraction of the initial candidate center set, $C_{\mathrm{cand}}$, is mathematically formulated as:

(9) $$\begin{array}{cc} C_{\mathrm{cand}}= & \left\{(3x_{d}+1,3y_{d}+1\right)\notin \Omega_{\mathrm{invalid}}\;|\\ \; & \;\max_{i,j\in \{0,1,2\}}B_{\mathrm{cand}}\left(3x_{d}+i,3y_{d}+j\right)=1\} \end{array}$$

where $\left(x_{d},y_{d}\right)$ denotes the downsampled grid coordinates; the constraint $\notin \Omega_{\mathrm{invalid}}$ ensures that the remapped candidate coordinates strictly avoid the predefined global invalid regions; and the constant offset of +1 serves to anchor these coordinates to the geometric center of each activated cell. Subsequently, this sparsified candidate set is forwarded for classification and target decoupling.

The pseudocode for the proposed MAFF strategy is summarized in Algorithm 1.
**Algorithm 1:** Motion and Appearance Feature Fusion Strategy**Input**   **:** Current frame $I_{t}$ and sequence of historical frames ${\left\{I_{t-k}\right\}}_{k=1}^{K}$;      Hyperparameters $\alpha ,T_{m},\tau_{\mathrm{diff}}$.**Output:** Coarse candidate center set $C_{\mathrm{cand}}$. **1** $\left\{{\tilde{I}}_{t-k}\right\},\Omega_{\mathrm{invalid}}\leftarrow \mathrm{SpatialRegistration}\left({\left\{I_{t-k}\right\}}_{k=1}^{K},I_{t}\right)$ **2** $\left\{{\hat{I}}_{t-k}\right\}\leftarrow \mathrm{PhotometricCompensation}\left(\left\{{\tilde{I}}_{t-k}\right\},I_{t}\right)$ **3** $B_{t}\leftarrow {\mathrm{Median}}_{1\leq k\leq K}\left(\left\{{\hat{I}}_{t-k}\right\}\right)$ **4** $D_{t}\leftarrow \left|I_{t}-B_{t}\right|$ **5** $\left(u,v\right)\leftarrow \mathrm{DenseOpticalFlow}\left(I_{t},{\hat{I}}_{t-1}\right)$ **6** $M_{t}\leftarrow N\left(G_{\mathrm{flow}}\left(\sqrt{u^{2}+v^{2}}\right)\right)$ **7** $D_{t}^{\prime }\leftarrow \mathrm{EnhanceDifference}\left(D_{t},M_{t},T_{m},\alpha ,\Omega_{\mathrm{invalid}}\right)$ **8** $B_{\mathrm{cand}}\leftarrow I\left(D_{t}^{\prime }\geq \tau_{\mathrm{diff}}\right)$ **9** $C_{\mathrm{cand}}\leftarrow \mathrm{GridNMS}\left(B_{\mathrm{cand}},\Omega_{\mathrm{invalid}}\right)$**10** **return** $C_{\mathrm{cand}}$

### 3.3. Spatial Attention-Guided Target Decoupling Module

Although the front-end perception stage captures moving pixels, the resulting candidate regions frequently exhibit severe spatial overlap among targets [24]. To resolve this challenge, we propose the SA-TD module. This module transitions the detection task from conventional coarse-grained bounding box regression to fine-grained heatmap decoding, thereby enabling the decoupling of densely clustered targets within the deep feature space.

#### 3.3.1. Candidate Screening via Binary Classification

To eliminate false positives induced by parallax effects and dynamic background clutter, we initially employ a lightweight binary classifier to serve as a spatiotemporal filter [15]. Let $C_{\mathrm{cand}}={\left\{c_{i}\right\}}_{i=1}^{N_{c}}$ denote the set of coarse candidate centers derived from the preceding background subtraction stage. For each candidate center $c_{i}$, we crop localized patches from the current frame *t* and three consecutive historical templates (i.e., t−1, t−2, and t−3) to construct a spatiotemporal input tensor, $X_{i}^{\mathrm{bin}}\in R^{4\times 21\times 21}$. Ultimately, the probability of genuine target presence, $p_{i}$, is predicted by the binary classification network $F_{\mathrm{binary}}$:

(10) $$p_{i}=F_{\mathrm{binary}}\left(X_{i}^{\mathrm{bin}};\theta_{\mathrm{bin}}\right)$$

where $\theta_{\mathrm{bin}}$ denotes the learnable parameters of the network. To suppress evident background clutter while preserving potential challenging targets, we establish a confidence threshold, $\tau_{\mathrm{bin}}$, to conduct a preliminary screening of the initial candidate set:

(11) $$C_{\mathrm{pending}}=\left\{c_{i}\in C_{\mathrm{cand}}\;|\;p_{i}\geq \tau_{\mathrm{bin}}\right\}$$

Following this lightweight filtering stage, the retained candidate set, $C_{\mathrm{pending}}$, is forwarded to the subsequent multi-target heatmap regression network.

#### 3.3.2. Spatial Attention-Guided Heatmap Generation

To establish a unified mathematical formulation for the network architecture, we initially define a generalized dual-convolutional base operator, denoted as $\Phi_{m}\left(\cdot \right)$. For any given input feature tensor F, its strictly cascaded expansion is mathematically defined as:

(12) $$\begin{array}{cc} \Phi_{m}\left(F\right) & =\mathrm{ReLU}(\mathrm{BN}({\mathrm{Conv}}_{m}^{3\times 3}(\\ \; & \;\mathrm{ReLU}(\mathrm{BN}\left({\mathrm{Conv}}_{m}^{3\times 3}\left(F\right)\right))))) \end{array}$$

where *m* denotes the output channel dimension of the module; ${\mathrm{Conv}}_{m}^{3\times 3}$ represents a 3×3 convolutional operator for feature mapping that yields *m* output channels; BN signifies the batch normalization operation; and ReLU corresponds to the Rectified Linear Unit non-linear activation function.

With the base operator established, for each candidate point $c_{i}\in C_{\mathrm{pending}}$, we extract a localized spatiotemporal patch of 45×45 pixels to formulate the regression input tensor, $X^{\mathrm{reg}}\in R^{4\times 45\times 45}$. This tensor is subsequently processed through two cascaded feature extraction stages—each equipped with a 2×2 spatial max pooling operation ($M_{↓}$)—and is ultimately projected to yield the mid-level feature map $F_{\mathrm{mid}}\in R^{64\times 11\times 11}$:

(13) $$F_{\mathrm{mid}}=M_{↓}\left(\Phi_{64}\left(M_{↓}\left(\Phi_{32}\left(X^{\mathrm{reg}}\right)\right)\right)\right)$$

To accentuate the subtle motion signatures of tiny targets amidst complex background clutter, we integrate a spatial attention module within the mid-level feature space [25]. Specifically, we initially derive two-dimensional spatial descriptors by applying average pooling ($P_{\mathrm{avg}}$) and max pooling ($P_{\max}$) operations across the channel axis:

(14) $$F_{\mathrm{avg}}^{s}=P_{\mathrm{avg}}\left(F_{\mathrm{mid}}\right),\;F_{\max}^{s}=P_{\max}\left(F_{\mathrm{mid}}\right)$$

where $F_{\mathrm{avg}}^{s}\in R^{1\times 11\times 11}$ denotes the average-pooled feature descriptor, which aggregates global statistical information; and $F_{\max}^{s}\in R^{1\times 11\times 11}$ represents the max-pooled feature descriptor, which captures the most salient local motion responses. By collapsing the channel dimension, these paired descriptors effectively steer the network’s attention toward spatial regions exhibiting prominent motion signatures.

These descriptors are concatenated along the channel axis and subsequently processed through a 7×7 convolutional layer, ${\mathrm{Conv}}_{1}^{7\times 7}$, coupled with a Sigmoid activation function to derive the spatial attention weight map $A_{s}\in R^{1\times 11\times 11}$:

(15) $$A_{s}=\mathrm{Sigmoid}\left({\mathrm{Conv}}_{1}^{7\times 7}\left(\left[F_{\mathrm{avg}}^{s};F_{\max}^{s}\right]\right)\right)$$

where ${\mathrm{Conv}}_{1}^{7\times 7}$ denotes a spatial convolutional operator featuring a 7×7 kernel and yielding a single output channel; and [·;·] represents the channel-wise feature concatenation operation.

Subsequently, the attention mask $A_{s}$ is element-wise multiplied with the mid-level feature map $F_{\mathrm{mid}}$, and the resulting tensor is directly propagated to the high-order feature extraction stage. By employing the previously defined generalized operator $\Phi_{m}\left(\cdot \right)$, these saliency-enhanced features are further projected into high-order semantic representations, yielding $F_{\mathrm{high}}\in R^{128\times 11\times 11}$:

(16) $$F_{\mathrm{high}}=\Phi_{128}\left(F_{\mathrm{mid}}⊗A_{s}\right)$$

where ⊗ denotes the spatial element-wise multiplication operation.

In contrast to conventional networks that regress a single coordinate offset, the SA-TD module decodes the high-order semantic representations, $F_{\mathrm{high}}$, into a spatial probability distribution to effectively decouple multiple densely clustered targets within the localized patch. Specifically, the flattened high-order feature tensor is mapped to a probability vector via a Multi-Layer Perceptron and subsequently reshaped to form a coarse local heatmap, ${\hat{H}}_{\mathrm{coarse}}\in {\left[0,1\right]}^{15\times 15}$:

(17) $$\begin{array}{cc} {\hat{H}}_{\mathrm{coarse}} & =R_{\mathrm{reshape}}^{15\times 15}(\mathrm{Sigmoid}(W_{225}\\ & \;\mathrm{ReLU}\left(\mathrm{BN}(W_{512}\;\mathrm{Flatten}\left(F_{\mathrm{high}}\right))\right))) \end{array}$$

where Flatten(·) denotes the operation that flattens a three-dimensional feature tensor into a one-dimensional vector; $W_{512}$ and $W_{225}$ represent the weight matrices of the fully connected layers, projecting the features into 512 and 225 dimensions, respectively; and $R_{\mathrm{reshape}}^{15\times 15}\left(\cdot \right)$ signifies the spatial reshaping operator that rearranges the resulting 225-dimensional probability vector into a 15×15 two-dimensional grid.

#### 3.3.3. Optimization Objective and Step-Degradation Inference

During the training phase, given a localized patch encompassing $N_{p}$ genuine moving targets, let ${\left\{\mu_{j}\right\}}_{j=1}^{N_{p}}$ denote their corresponding relative center coordinates within the local patch coordinate frame. The ground-truth heatmap, H, for this specific patch is subsequently synthesized by rendering two-dimensional Gaussian kernels:

(18) $$H\left(x\right)=\max_{1\leq j\leq N_{p}}\exp\left(-\frac{{∥x-\mu_{j}∥}_{2}^{2}}{2\sigma_{p}^{2}}\right)$$

where $x={\left[x,y\right]}^{T}$ denotes the two-dimensional spatial coordinate vector within the localized patch; $\mu_{j}={\left[\mu_{x_{j}},\mu_{y_{j}}\right]}^{T}$ represents the relative center coordinates of the *j*-th target; and $\sigma_{p}$ signifies the standard deviation parameter governing the spatial extent of the Gaussian kernel.

To model the spatial distribution of targets, the network is optimized via the Mean Squared Error loss function:

(19) $$L_{\mathrm{reg}}=\frac{1}{N_{r}}\sum_{x}{∥{\hat{H}}_{\mathrm{coarse}}\left(x\right)-H\left(x\right)∥}_{2}^{2}$$

where $N_{r}$ denotes the total number of spatial pixels comprising the coarse heatmap.

During the inference phase, the predicted coarse heatmap is bilinearly upsampled to the original localized patch resolution of 45×45, yielding the refined heatmap ${\hat{H}}_{\mathrm{fine}}$. To decouple and extract tiny targets exhibiting severe spatial overlap within densely clustered scenes—and to effectively mitigate the missed detection issue induced by the mutual suppression of adjacent genuine targets—we introduce a step-threshold degradation algorithm founded on topological connected components.

Specifically, we define a monotonically decreasing discrete threshold sequence, $T=\{\tau_{0},\tau_{1},\ldots ,\tau_{S}\}$, where *S* represents the total number of degradation steps. The initial threshold is initialized as $\tau_{0}=\max\left({\hat{H}}_{\mathrm{fine}}\right)$ and is iteratively decayed by a constant step size of Δτ until reaching a predefined lower bound, $\tau_{\min}$.

During the *s*-th iteration step (s∈{0,1,…,S}) associated with the specific threshold $\tau_{s}$, we extract the set of independent connected components, denoted as $O^{\left(s\right)}=\left\{o_{j}^{\left(s\right)}\right\}$, wherein the refined heatmap satisfies the condition ${\hat{H}}_{\mathrm{fine}}\geq \tau_{s}$. The spatial coordinates of the target candidates are subsequently determined by identifying the local maxima within each respective connected component. This yields the candidate set for the current iteration, $V_{s}$, formulated as:

(20) $$V_{s}=\{\arg\max_{x\in o_{j}^{\left(s\right)}}{\hat{H}}_{\mathrm{fine}}\left(x\right)\;|\;o_{j}^{\left(s\right)}\in O^{\left(s\right)}\}$$

To preclude the redundant extraction of identical salient targets across varying threshold levels, we introduce an inter-step exclusion mechanism designed to dynamically maintain a global set of valid coordinates, D. Initializing with $D_{0}=V_{0}$, the subsequent updates to this global set strictly adhere to a set difference operational rule:

(21) $$D_{s}=D_{s-1}\cup \left(V_{s}\setminus D_{s-1}\right)$$

This exclusion operation (∖) prevents previously detected coordinates from being repeatedly added across different threshold levels. Upon reaching the terminal threshold, $\tau_{S}$, the resulting set, $D_{S}$, provides the spatial coordinates of the separated densely clustered targets within the localized patch.

**Figure 4:**
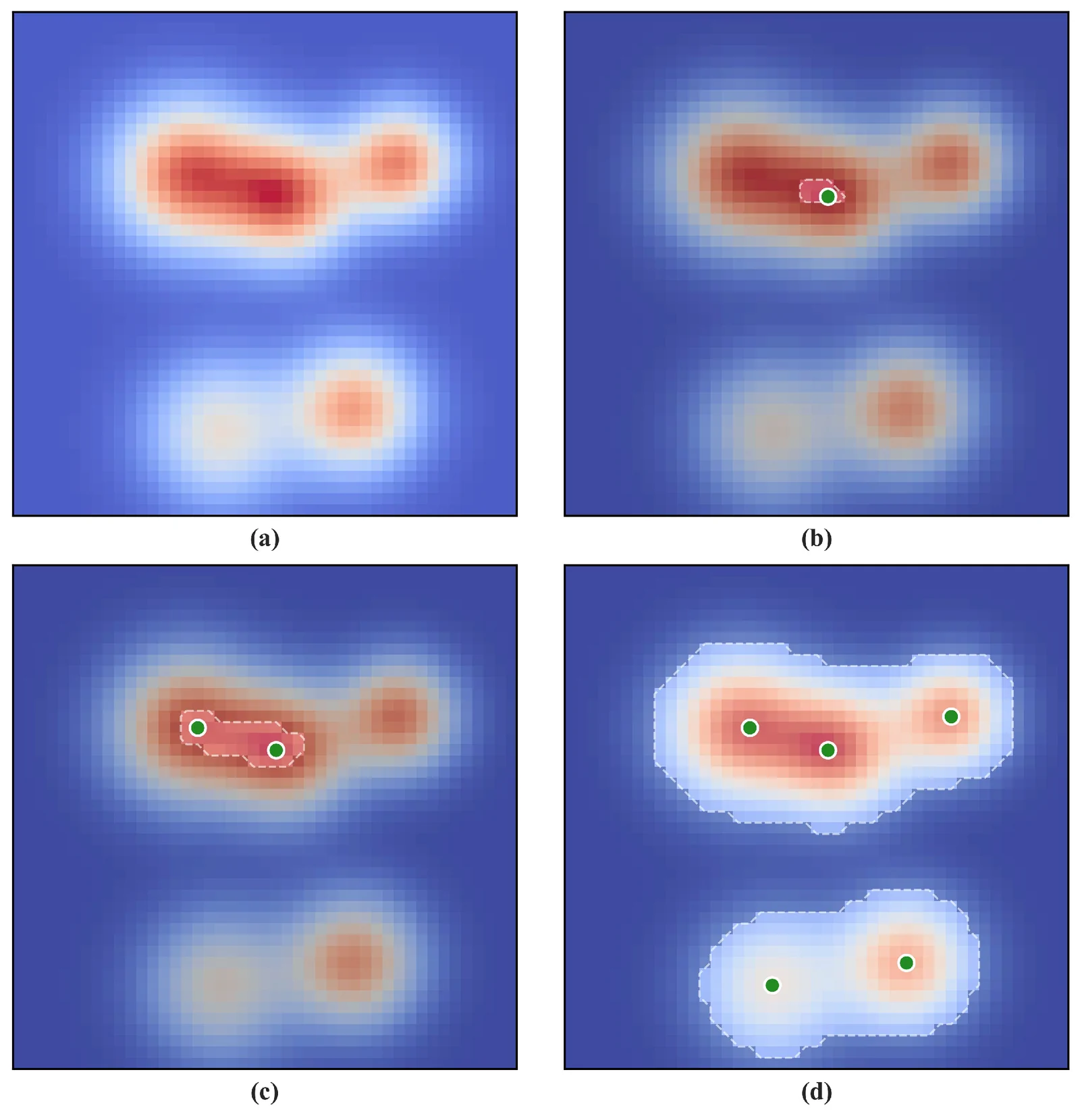
Step-threshold target decoupling process. (**a**) Original heatmap (${\hat{H}}_{\mathrm{fine}}$). (**b**–**d**) Progressive extraction at decreasing thresholds $\tau_{s}$ (dashed outlines: connected domains; green dots: target centers). (**b**) $\tau_{1}=0.97$: most salient target extraction. (**c**) $\tau_{7}=0.90$: domain expansion for additional targets. (**d**) $\tau_{\min}=0.17$: final target separation via cross-layer exclusion.

To illustrate this procedure, Figure 4 shows the progressive target decoupling process. Starting from the refined heatmap ${\hat{H}}_{\mathrm{fine}}$ (Figure 4a), a high initial threshold ($\tau_{1}=0.97$, Figure 4b) extracts the most salient targets as independent connected components. As τ gradually decreases ($\tau_{7}=0.90$, Figure 4c), additional weaker target responses progressively emerge. At the terminal threshold ($\tau_{\min}=0.17$, Figure 4d), further target responses are separated, while the inter-step exclusion mechanism reduces redundant detections across different threshold levels.

The pseudocode for the proposed SA-TD module is summarized in Algorithm 2.
**Algorithm 2:** Spatial Attention-Guided Target Decoupling Module
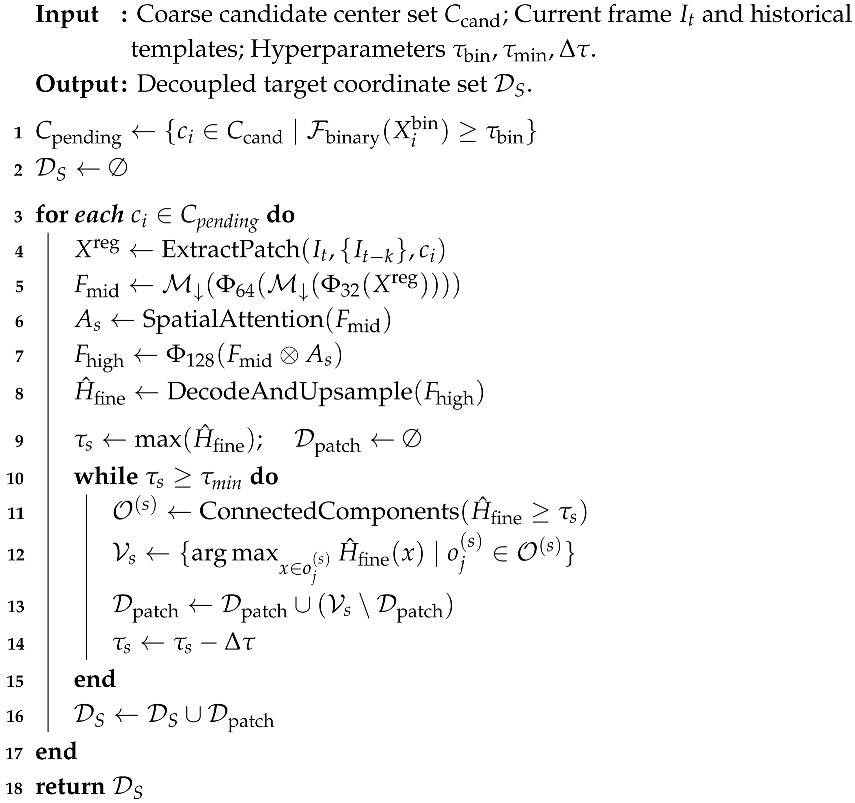


### 3.4. Scene-Prior Guided Filtering Mechanism

Although SA-TD effectively separates densely clustered target responses, candidate-level features alone remain insufficient to suppress false alarms caused by building parallax, registration residuals, vegetation motion, and other structured background interference [26]. We therefore introduce the SPGF mechanism to provide a soft scene-level constraint for candidate verification. SPGF estimates vehicle-compatible scene responses directly from visual context and generates a continuous probability prior for suppressing contextually implausible detections. Unlike rigid binary masks constructed from external geographic data such as OpenStreetMap (OSM) [27], the learned scene prior does not require precise map-to-image registration and provides soft contextual constraints that reduce the risk of discarding valid targets near uncertain or transitional regions.

#### 3.4.1. Formulation of the Network Topology

SPGF reuses the temporally aggregated reference background $B_{t}$ constructed in MAFF as its scene-context input. Since $B_{t}$ is obtained from spatially and photometrically aligned historical frames through temporal median filtering, transient foreground responses are attenuated while relatively stable scene structures are retained. The network adopts a fully convolutional residual encoder–decoder architecture to extract contextual features relevant to vehicle accessibility and motion activity. The encoder progressively captures multi-scale representations, while an atrous spatial pyramid enlarges the effective receptive field at the bottleneck [28]. The decoder then progressively restores spatial resolution through bilinear upsampling and skip connections.

For compact notation, let ${\mathrm{CGS}}_{n}^{k,r}\left(\cdot \right)$ denote a k×k convolution with *n* output channels and dilation rate *r*, followed by Group Normalization (GN) [29] and Swish activation [30]. Moreover, $\Psi_{n}^{\delta }\left(\cdot \right)$ denotes a residual block with *n* output channels and stride δ, where a 1×1 projection is applied to the shortcut when the feature dimensions change. The number of output channels at the *l*-th encoder stage is defined as $n_{l}=24\times 2^{l-1}$, l=1,…,4, with $\delta_{1}=1$ and $\delta_{l}=2$ for l=2,…,4.

The complete network is formulated recursively as

(22) $$\left\{\begin{array}{l} E_{0}={\mathrm{CGS}}_{n_{1}}^{3,1}\left(B_{t}\right),\\ E_{l}=\Psi_{n_{l}}^{\delta_{l}}\left(E_{l-1}\right),\;l=1,\ldots ,4,\\ Z={\mathrm{CGS}}_{n_{4}}^{1,1}\left([{\mathrm{CGS}}_{n_{3}}^{1,1}\left(E_{4}\right),{\mathrm{CGS}}_{n_{3}}^{3,2}\left(E_{4}\right),{\mathrm{CGS}}_{n_{3}}^{3,4}\left(E_{4}\right),{\mathrm{CGS}}_{n_{3}}^{3,8}\left(E_{4}\right)]\right),\\ U_{4}=Z,\\ U_{l}={\mathrm{CGS}}_{n_{l}}^{3,1}([{\mathrm{Up}}_{2}(U_{l+1})E_{l}]),\;l=3,2,1,\\ {}[P_{\mathrm{acc}},P_{\mathrm{mot}}]=\mathrm{Sigmoid}({\mathrm{Conv}}_{2}^{1\times 1}(U_{1})). \end{array}\right.$$

where $E_{l}$ and $U_{l}$ denote the encoder and decoder features at the *l*-th scale, respectively, Z denotes the bottleneck feature obtained through multi-scale atrous aggregation, and ${\mathrm{Up}}_{2}\left(\cdot \right)$ denotes bilinear upsampling by a factor of two. At the bottleneck, parallel convolutions with dilation rates {2,4,8} aggregate broader scene context while preserving local structural information. The decoder progressively fuses the bottleneck representation with encoder features and produces two complementary probability maps: $P_{\mathrm{acc}}$ characterizes vehicle-accessibility compatibility, whereas $P_{\mathrm{mot}}$ characterizes vehicle-motion compatibility.

#### 3.4.2. Network Optimization Objective

The supervision maps are constructed from temporally aggregated vehicle trajectories rather than from a coarse binary scene mask. For each training anchor, trajectory observations from the preceding 20 frames are aligned to the current reference frame. The accessibility branch uses all valid trajectory points, whereas the motion branch uses only trajectory points associated with displacements of at least 0.8 m. Each trajectory point is represented by a localized Gaussian response with a radius of 10 pixels and a standard deviation of 5 pixels at the native image resolution, yielding two soft supervision maps for vehicle accessibility and motion activity, respectively.

The two prediction branches are jointly optimized using spatially weighted variants of focal loss [31] and Dice loss [32]. Specifically, $L_{\mathrm{focal}}^{\mathrm{acc}}$ and $L_{\mathrm{focal}}^{\mathrm{mot}}$ emphasize hard-to-classify pixels and reduce the dominance of easy background samples in the vehicle-accessibility and vehicle-motion branches, respectively. Meanwhile, $L_{\mathrm{dice}}^{\mathrm{acc}}$ and $L_{\mathrm{dice}}^{\mathrm{mot}}$ encourage spatial consistency between the predicted and ground-truth prior maps. The overall objective is formulated as:

(23) $$L_{\mathrm{SPGF}}=\left(L_{\mathrm{focal}}^{\mathrm{acc}}+0.5L_{\mathrm{dice}}^{\mathrm{acc}}\right)+0.7\left(L_{\mathrm{focal}}^{\mathrm{mot}}+0.5L_{\mathrm{dice}}^{\mathrm{mot}}\right).$$

The coefficient 0.5 balances the region-level Dice constraint with the pixel-wise focal objective, while the coefficient 0.7 balances the contribution of motion-activity supervision relative to vehicle-accessibility supervision.

#### 3.4.3. Prior Refinement and Filtering

During inference, the two complementary predictions are first fused through their geometric mean:

(24) $$P_{\mathrm{scene}}=\sqrt{P_{\mathrm{acc}}⊙P_{\mathrm{mot}}},$$

where ⊙ denotes element-wise multiplication. This fusion assigns high prior confidence to regions jointly supported by vehicle-accessibility and vehicle-motion cues.

Although $P_{\mathrm{scene}}$ provides a continuous scene-level constraint, isolated responses and short discontinuities may remain in the predicted map. We therefore apply a lightweight topology-preserving refinement $R_{\mathrm{topo}}\left(\cdot \right)$, consisting of Gaussian smoothing, weak- and strong-confidence support extraction, multi-directional morphological closing, connected-component filtering according to the presence of strong-confidence support, area, and elongation, and soft attenuation of unreliable responses. The overall refinement process is compactly formulated as:

(25) $$P_{\mathrm{prior}}=R_{\mathrm{topo}}\left(P_{\mathrm{scene}}\right).$$

This refinement bridges short discontinuities and suppresses isolated or topologically inconsistent responses. Unlike binary scene masks, the refinement retains continuous confidence values, allowing uncertain contextual evidence near structural boundaries to be preserved rather than converted into hard binary decisions.

The resulting prior $P_{\mathrm{prior}}$ is finally applied to the target set $D_{S}$ produced by SA-TD:

(26) $$D_{\mathrm{final}}=\left\{p\in D_{S}\;|\;P_{\mathrm{prior}}\left(p\right)\geq \tau_{\mathrm{prior}}\right\}.$$

where $\tau_{\mathrm{prior}}$ denotes the scene-prior confidence threshold.

Thus, SPGF acts as a soft contextual filter rather than an independent target detector, suppressing detections inconsistent with the inferred scene context while preserving the fine-grained localization produced by SA-TD.

The pseudocode of the proposed SPGF mechanism is summarized in Algorithm 3.
**Algorithm 3:** Scene-Prior Guided Filtering Mechanism
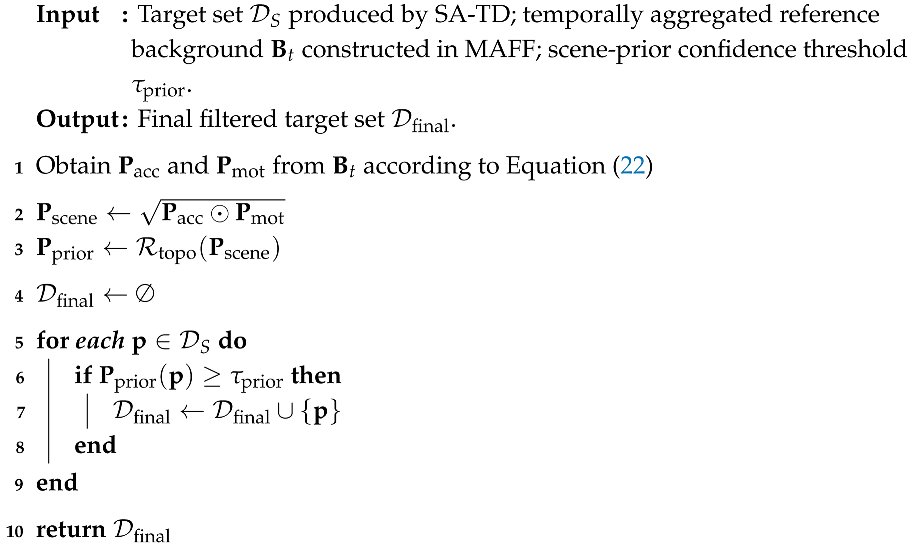


## 4. Experimental Setup

### 4.1. Datasets and Evaluation Metrics

We evaluate MDF-Det on the WPAFB 2009 dataset [8], a standard WAMI benchmark acquired at 1.25 Hz using a camera system composed of six optical sensors. The individual sensor images are spatially stitched into gigapixel-scale mosaics covering an area of approximately 35 km^2^. The dataset contains 1025 frames and is divided into a 512-frame training sequence and a 513-frame test sequence. The binary classification and heatmap regression networks in SA-TD, together with the scene-prior network in SPGF, are trained exclusively using samples constructed from the training sequence, while the test sequence is reserved solely for final performance evaluation.

The dataset presents substantial challenges for moving-target detection, including frame resolutions exceeding 315 megapixels, dense traffic with approximately 2000 vehicles per frame, and extremely small targets with an average size of approximately 9×18 pixels. Following the commonly adopted evaluation protocol [11], quantitative evaluation is conducted on six representative AOIs, namely AOIs 01, 02, 03, 34, 40, and 41.

For qualitative cross-scene evaluation of SPGF, representative scenes from the Greene 2007 dataset [33] are additionally used. Greene 2007 is used exclusively for zero-shot inference and is not involved in training, validation, model selection, or hyperparameter tuning.

Detection performance is quantitatively assessed using Recall, Precision, and the $F_{1}$ score [11]. A predicted target location is considered a true positive if it is within 20 pixels of the corresponding ground-truth location. Computational efficiency is further evaluated in terms of the number of trainable parameters, model size, FLOPs per frame, peak GPU memory, and inference time in seconds per frame. Runtime contribution is additionally reported to characterize the relative computational cost of each module.

### 4.2. Baseline Methods and Implementation Details

To evaluate the proposed approach, MDF-Det is compared with five representative baseline methods, including ClusterNet [11], the CNN-based method of Zhou et al. [15], the heatmap-regression-based HMRN [17] and HM-Net [6], and CATLoss [34]. Since official implementations directly applicable to our experimental setting are unavailable for these methods, all baselines are independently reimplemented according to the architectures, training procedures, and parameter settings described in their original publications. The principal implementation and training settings of the reproduced baselines are summarized in Table 1. ClusterNet, HMRN, HM-Net, and CATLoss are trained from random initialization, whereas CNNs are initialized from the publicly released weights of the reference implementation and subsequently retrained on the adopted training partition. To ensure a fair comparison, all reproduced baselines and MDF-Det use the same training, validation, and test data partitions, as well as the same evaluation protocol. Model selection and hyperparameter tuning are performed exclusively on the validation set, after which the selected configurations are fixed and evaluated on the test set without further adjustment. Therefore, all baseline results reported in this study are obtained from our reproduced implementations rather than directly adopted from the original publications.

The MDF-Det pipeline is implemented using PyTorch and TensorFlow/Keras. All experiments are conducted on a workstation equipped with an Intel Core i9-14900K CPU and an NVIDIA RTX 3090 GPU. All methods are evaluated on the same six AOIs under an identical detection matching criterion and evaluation procedure.

The hyperparameters of MDF-Det are set as follows. For MAFF, we use K=5, $T_{m}=0.1$, α=15, and $\tau_{\mathrm{diff}}=1$. For SA-TD, we set $\tau_{\mathrm{bin}}=0.7$, Δτ=0.01, and $\tau_{\min}=0.17$. For SPGF, the scene-prior confidence threshold is set to $\tau_{\mathrm{prior}}=0.2$. These hyperparameters are selected using the temporally separated validation set and are subsequently fixed for all test-set evaluations unless otherwise specified.

### 4.3. Network Training Details

The binary classification network used for candidate screening in SA-TD follows the network architecture of Zhou et al. [15], but all model parameters are retrained in this study rather than using the pretrained weights from the original method. The binary classification, heatmap regression, and SPGF networks are all trained and validated using temporally separated and non-overlapping intervals constructed exclusively from the 512-frame training sequence: frames 100–379 are used for training, whereas frames 400–499 are reserved for validation. No random frame-level splitting is performed, thereby reducing temporal correlation between adjacent WAMI frames across the training and validation subsets. The 513-frame test sequence is reserved exclusively for final evaluation and is not involved in model training, validation, model selection, or hyperparameter tuning.

For the binary classifier, 3,600,000 training samples and 36,000 validation samples are constructed as four-frame spatiotemporal patches of size 4×21×21 from candidate locations in the corresponding training and validation intervals. For the heatmap regression network in SA-TD, 2,824,620 training samples and 696,090 validation samples are constructed as four-frame spatiotemporal patches of size 4×45×45, with each target label represented by a 15×15 heatmap. A mean spatiotemporal patch, estimated from at most 50,000 training samples, is subtracted from the inputs for normalization. The heatmap regression network is trained for 100 epochs using Adam with an initial learning rate of $1\times 10^{-4}$, a batch size of 256, and the mean squared error loss.

For SPGF, 5133 training samples and 1766 validation samples are independently constructed from the same temporally separated intervals using a sliding-window strategy on the original-resolution temporal reference background $B_{t}$. Specifically, 384×384 patches and their corresponding trajectory-supervised probability maps are extracted without resizing the full image to a fixed low resolution. For each anchor frame, up to 96 patches are sampled using a 40:30:30 ratio of trajectory-centered positive samples, high-texture hard negatives, and random negatives, respectively. Negative samples are maintained at least 48 pixels from annotated trajectory points, while weak negative regions are assigned a reduced loss weight of 0.03 to account for the incompleteness of trajectory-based supervision.

**Figure 5:**
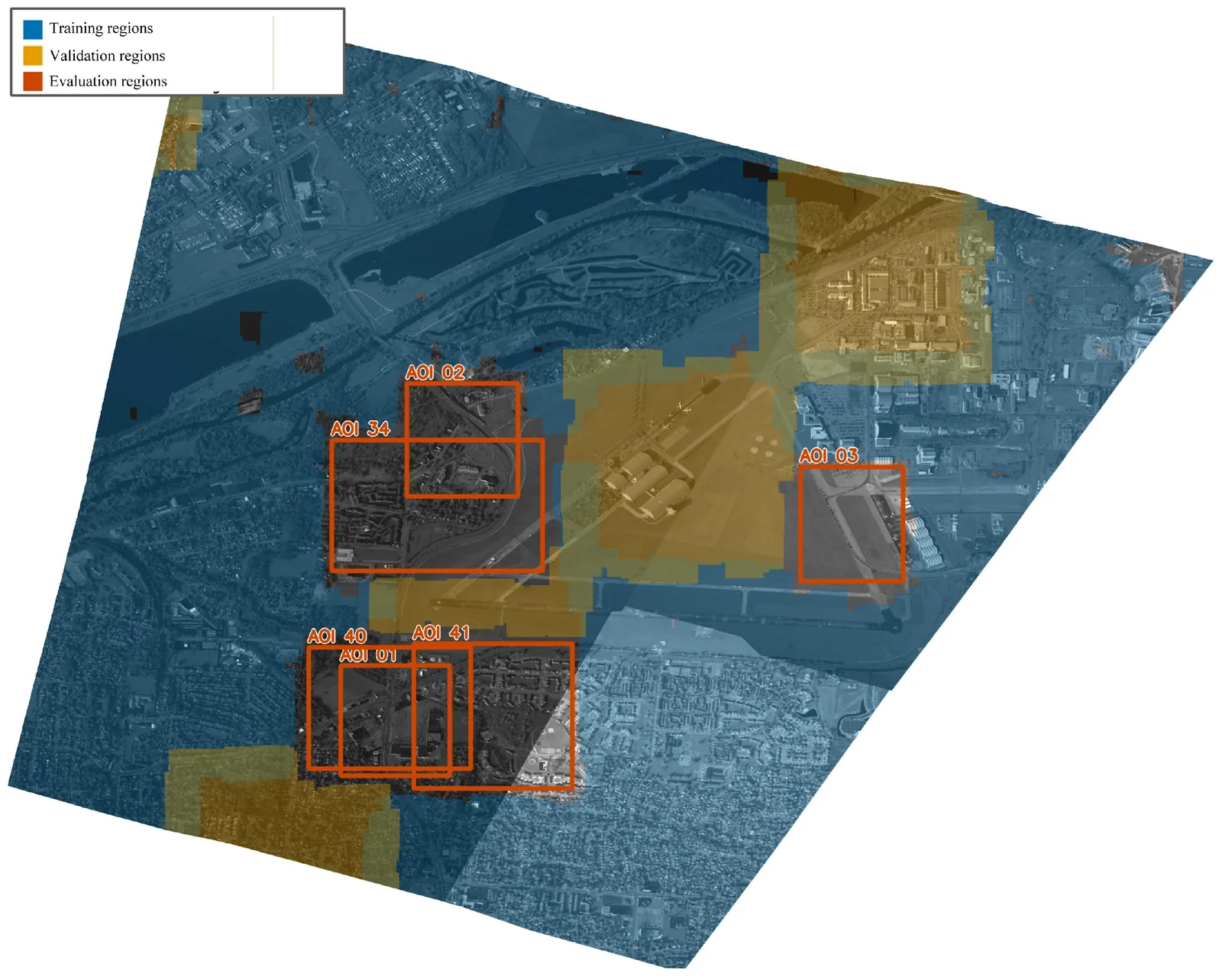
Spatial distribution of SPGF training, validation, and evaluation regions.

To prevent spatial information leakage, all trajectory annotations and image patches overlapping the six evaluation AOIs are excluded together with an additional 64-pixel safety margin. The resulting spatial partition of the training, validation, and evaluation regions is illustrated in Figure 5. During inference, the same 384×384 sliding-window strategy is applied to the full-resolution reference background, and predictions from overlapping windows are merged to reconstruct the scene-prior probability maps. For each fixed AOI, the full-resolution scene-prior map is constructed once before online frame-by-frame detection and is subsequently reused for candidate filtering. The SPGF network is trained for a maximum of 80 epochs using Adam with an initial learning rate of $2\times 10^{-4}$ and a batch size of 4, using the combined focal and Dice losses described above.

## 5. Results

### 5.1. Illustrative Operation

**Figure 6:**
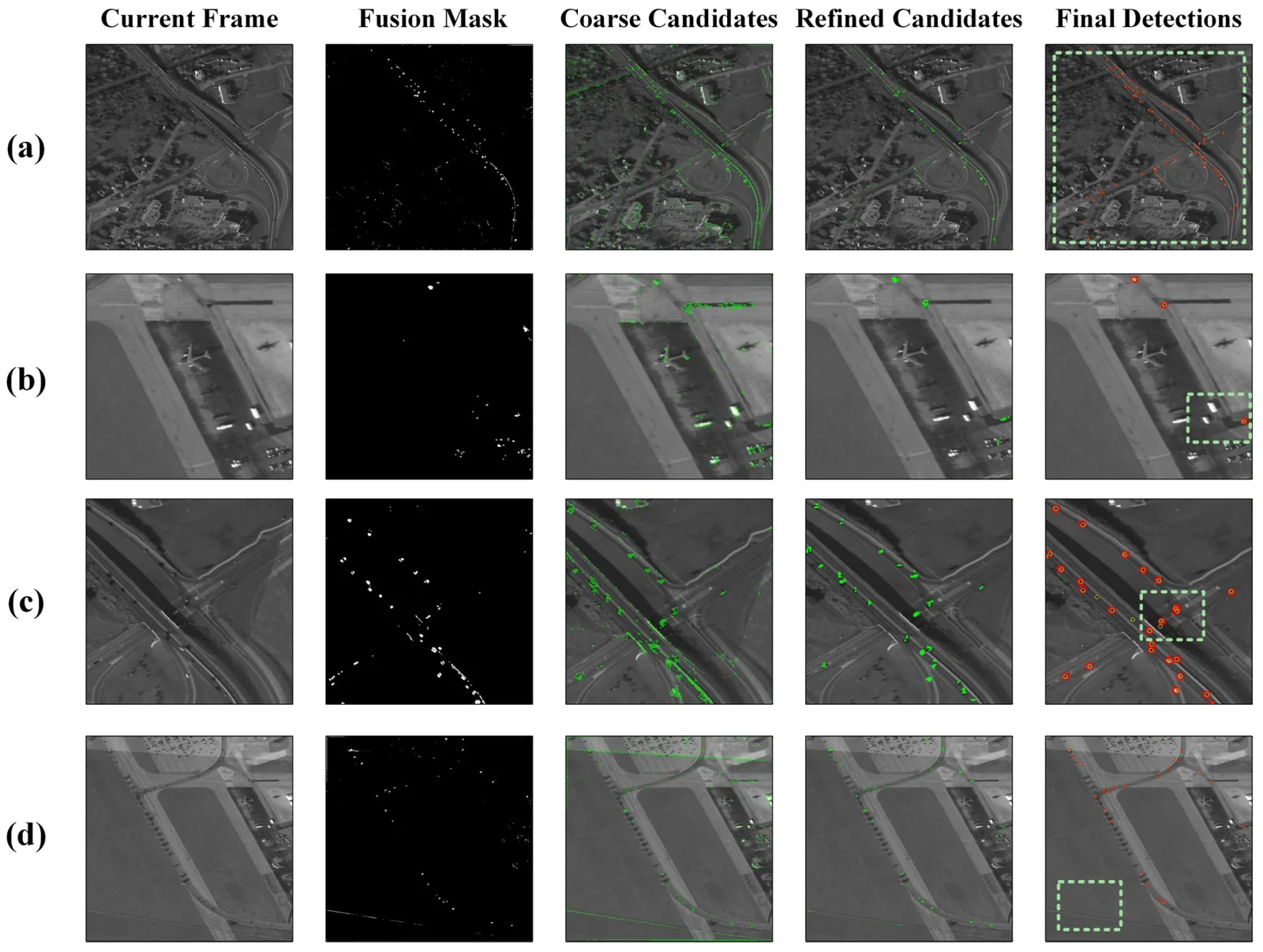
Qualitative visualization of the MDF-Det pipeline in four WAMI scenes: (**a**) curved intersection; (**b**) parking area; (**c**) multi-lane road; (**d**) sparse road. The columns show the current frame, fusion mask, coarse candidates, refined candidates, and final detections, respectively. Ground-truth locations and predictions are indicated by yellow dots and red circles, while green dashed boxes highlight challenging regions.

To qualitatively illustrate the operation of MDF-Det, Figure 6 presents representative intermediate results and final detections across four WAMI scenarios. Figure 6a shows the overall detection result on a full-scale scene, demonstrating that MDF-Det can maintain reliable target responses under an extremely low target-to-background ratio. The subsequent examples further illustrate the roles of the three cascaded modules: (1) **MAFF** enhances weak target responses by jointly exploiting motion and appearance cues, particularly for targets affected by shadows or weak motion contrast (Figure 6b); (2) **SA-TD** separates merged responses in densely distributed target regions, improving the discrimination of closely spaced targets (Figure 6c); and (3) **SPGF** suppresses false responses caused by registration residuals and structured background interference using the inferred scene-prior probability map (Figure 6d). The close correspondence between the predicted target locations (red circles) and the ground-truth annotations (yellow dots) further illustrates the ability of the proposed framework to localize moving targets while suppressing background-induced false alarms.

**Figure 7:**
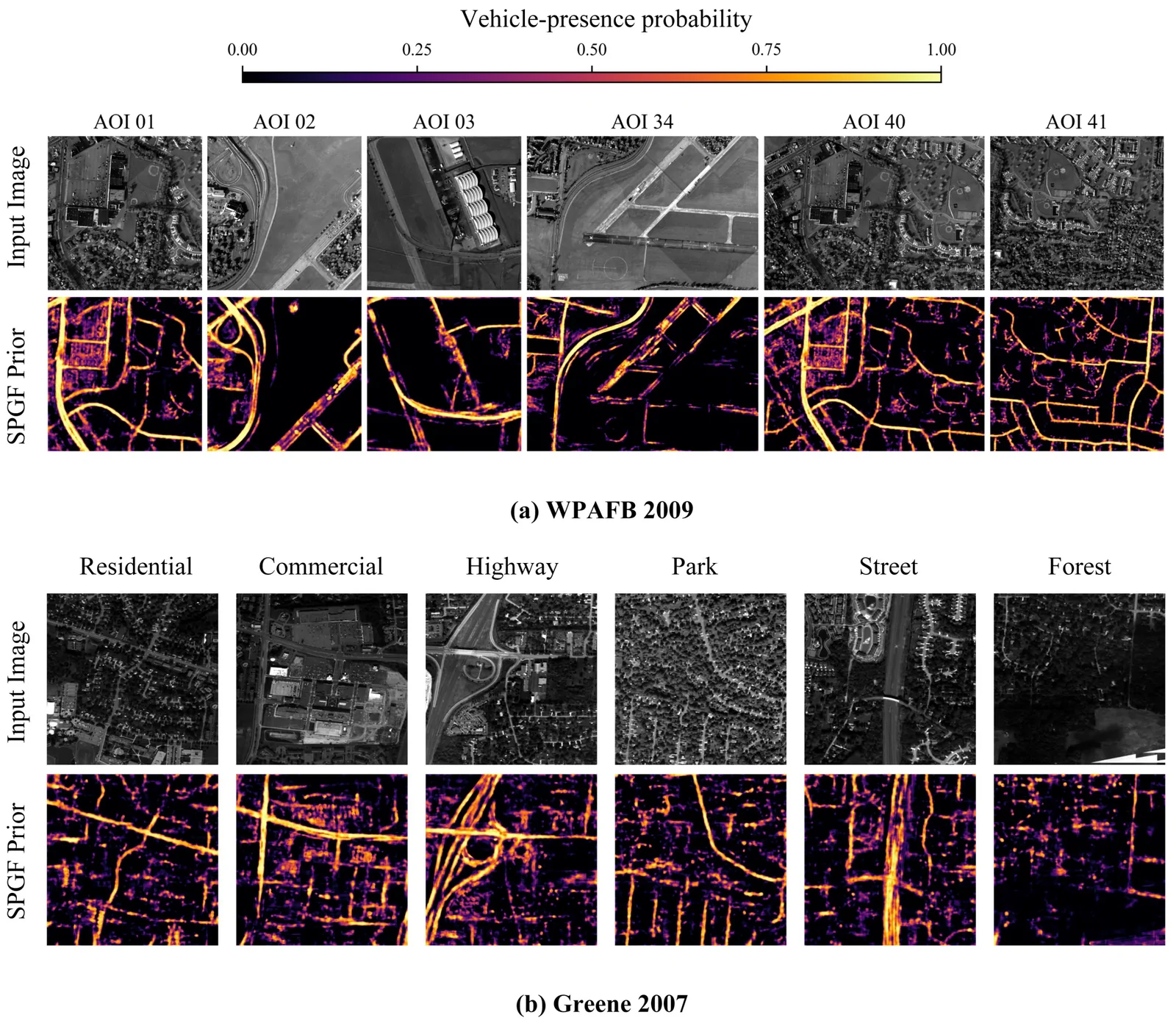
SPGF scene-prior probability maps on (**a**) WPAFB 2009 and (**b**) Greene 2007 under zero-shot transfer.

To evaluate the cross-scene generalization of SPGF, Figure 7 presents scene-prior probability maps on the six WPAFB 2009 evaluation AOIs and representative scenes from the Greene 2007 dataset. On WPAFB 2009, SPGF produces higher responses on vehicle-compatible structures such as roads, intersections, and parking areas, while assigning lower probabilities to most non-traffic regions. The model trained on WPAFB 2009 is further transferred directly to Greene 2007 without retraining, fine-tuning, or hyperparameter adjustment, where similar scene-aware response patterns are preserved across diverse environments. These results provide qualitative evidence that SPGF captures transferable scene-level compatibility rather than relying solely on scene-specific target distributions. Moreover, the inferred priors remain spatially selective: across the six WPAFB AOIs, the fraction of pixels with prior probability above 0.15 ranges from 23.40% to 48.41%, while only 12.02–24.90% of pixels exceed a probability of 0.50.

### 5.2. Comparison with Baseline Methods

Table 2 compares MDF-Det with five representative baseline methods across the six evaluation AOIs. MDF-Det achieves the highest $F_{1}$ score on all six AOIs, with an average performance of 0.878±0.043, compared with 0.832±0.071 for the strongest baseline, HM-Net. The corresponding mean $F_{1}$ improvement is 0.046 in absolute terms and 5.5% in relative terms. When compared with the best competing method on each individual AOI, MDF-Det yields absolute $F_{1}$ gains ranging from 0.013 to 0.048. The largest improvement is observed on AOI 03, where the $F_{1}$ score increases from 0.840 to 0.888. Improvements are also observed on AOIs 01 and 40, where the best baseline scores of 0.805 and 0.792 increase to 0.848 and 0.835, respectively. Overall, the results indicate that the performance improvement of MDF-Det is maintained across all six evaluated regions, while its cross-AOI standard deviation remains relatively low.

The reported mean and standard deviation characterize cross-region variability but do not directly quantify the uncertainty of the paired improvement over the strongest baseline. To further assess this uncertainty while accounting for temporal dependence between adjacent WAMI frames, we additionally perform a paired temporal-block bootstrap analysis between MDF-Det and HM-Net on the test sequence [35]. Under this additional temporal-block analysis, 10,000 paired bootstrap replicates are generated using non-overlapping 20-frame blocks, yielding an estimated paired $F_{1}$ improvement of 0.0828 with a 95% percentile confidence interval of [0.0766,0.0892]. Sensitivity analyses using 10- and 30-frame blocks yield confidence intervals of [0.0775,0.0874] and [0.0763,0.0887], respectively, demonstrating that the estimated paired improvement remains consistent across reasonable temporal block lengths.

### 5.3. Ablation Study

Table 3 evaluates the individual and complementary contributions of MAFF, SA-TD, and SPGF on AOI03. Among the individual modules, SA-TD provides the largest improvement, primarily by substantially increasing Recall, indicating its effectiveness in recovering and separating densely distributed target responses. In contrast, MAFF shifts the detection balance toward higher Precision, while SPGF improves Precision without reducing Recall, consistent with its role as a contextual false-alarm filter. More importantly, the modules exhibit clear complementarity: combining MAFF with SA-TD increases the $F_{1}$ score from 0.801 to 0.883, and further introducing SPGF improves Precision from 0.838 to 0.847 while preserving Recall at 0.933, yielding the best overall $F_{1}$ score of 0.888. These results indicate that MAFF improves candidate quality, SA-TD enhances target separability, and SPGF provides an additional scene-level constraint for false-alarm suppression.

Table 4 further examines the internal contributions of spatial attention and threshold degradation (TD) in SA-TD. When TD is disabled, a simple greedy peak-selection strategy is used instead, which iteratively selects the maximum response from the heatmap, removes the selected response, and retains the top 20 candidate locations. The results show that spatial attention provides the dominant improvement by markedly increasing Precision while maintaining high Recall, whereas TD further suppresses ambiguous heatmap responses and improves the Recall–Precision balance. Their combination achieves the highest $F_{1}$ score of 0.888, confirming the complementary roles of feature enhancement and adaptive target decoupling within SA-TD.

### 5.4. Hyperparameter Analysis

The main hyperparameters of MAFF, SA-TD, and SPGF are analyzed on the temporally separated validation set to characterize their effects on the Recall–Precision trade-off and overall detection behavior. The selected settings are then fixed for all subsequent test-set inference without further adjustment.

**Figure 8:**
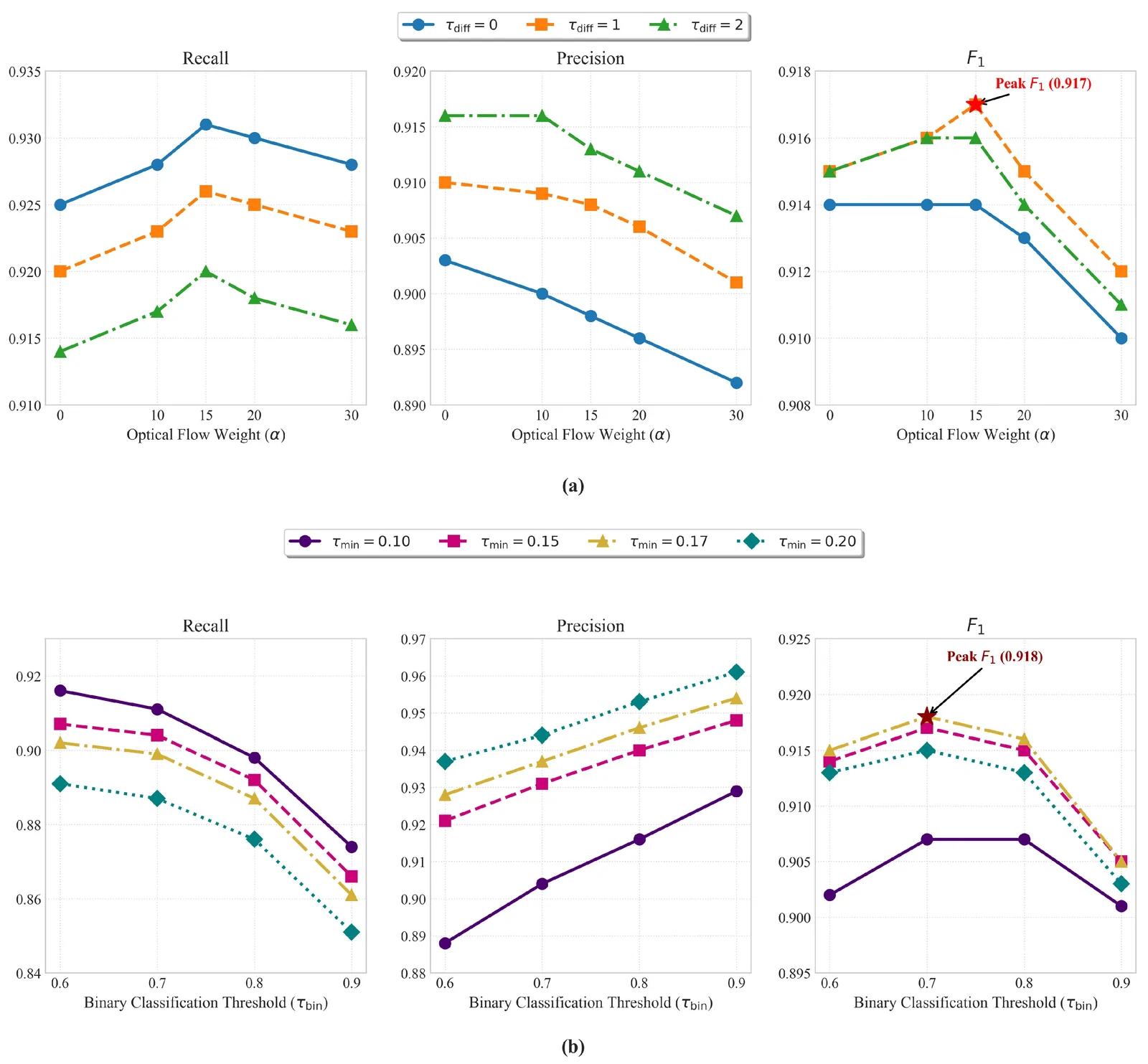
Validation-set hyperparameter sensitivity of (**a**) MAFF to α and $\tau_{\mathrm{diff}}$, and (**b**) SA-TD to $\tau_{\mathrm{bin}}$ and $\tau_{\min}$.

(1) MAFF benefits from moderate motion enhancement, while excessive enhancement introduces additional background responses. As shown in Figure 8a, increasing α initially improves Recall, whereas larger values reduce Precision and $F_{1}$. Increasing $\tau_{\mathrm{diff}}$ further shifts the operating point toward higher Precision and lower Recall. The $F_{1}$ score peaks at 0.917 when α=15 and $\tau_{\mathrm{diff}}=1$, and these values are therefore selected for subsequent test-set inference.

(2) SA-TD becomes increasingly precision-oriented as the screening thresholds increase, with intermediate settings remaining the most stable. Figure 8b shows that increasing $\tau_{\mathrm{bin}}$ or $\tau_{\min}$ generally reduces Recall while improving Precision, producing a unimodal $F_{1}$ trend. A stable high-performance region appears around $\tau_{\mathrm{bin}}=0.7$ and $\tau_{\min}=0.15$–0.17, with the maximum $F_{1}$ of 0.918 obtained at $\tau_{\mathrm{bin}}=0.7$ and $\tau_{\min}=0.17$; these values are therefore selected.

(3) SPGF progressively favors Precision as scene-prior filtering becomes stricter, while excessive filtering reduces overall performance. As shown in Table 5, increasing $\tau_{\mathrm{prior}}$ consistently lowers Recall and improves Precision, causing $F_{1}$ to first increase and then decline. The highest $F_{1}$ of 0.922 is obtained at $\tau_{\mathrm{prior}}=0.2$ for both $\tau_{\mathrm{bin}}=0.7$ and 0.9. Since $\tau_{\mathrm{bin}}=0.7$ retains higher Recall, $\tau_{\mathrm{prior}}=0.2$ and $\tau_{\mathrm{bin}}=0.7$ are selected for subsequent test-set inference.

### 5.5. Sensitivity to Localization Radius

To examine the influence of the localization criterion, we further evaluate MDF-Det using matching radii ranging from 5 to 25 pixels. All results are obtained from the same fixed detection outputs, with only the localization radius used for one-to-one matching being changed. Each ground-truth target is matched to at most one detection; unmatched detections and ground-truth targets are counted as false positives and false negatives, respectively.

As shown in Table 6, detection performance improves gradually as the localization radius increases, while the improvement becomes marginal at larger radii. Increasing the radius from 10 to 20 pixels improves the $F_{1}$ score by only 0.005, whereas a further increase to 25 pixels yields an additional gain of only 0.003. This relatively small variation indicates that MDF-Det is not highly sensitive to the localization radius and that the reported performance is not primarily attributed to the adopted 20-pixel matching criterion.

### 5.6. Computational Complexity and Runtime Analysis

Table 7 summarizes the computational complexity and runtime distribution of MDF-Det. The complete framework contains 10.794 M parameters, while its per-frame online neural-network inference requires 490.069 G FLOPs, with an overall runtime of 3.161 s/frame. FLOPs are reported for per-frame online neural-network inference: MAFF mainly consists of non-neural image-processing operations, whereas SPGF prior construction is performed once for each fixed AOI and is therefore excluded from the per-frame FLOP count. The binary classifier is the main computational bottleneck, accounting for 51.28% of the total runtime, mainly because it processes a large number of motion-generated candidate patches. In contrast, although SA-TD contains more parameters, it is applied only to a small subset of retained candidates, resulting in substantially lower FLOPs and runtime.

MAFF introduces no trainable parameters, with its computational cost mainly arising from multi-frame registration, motion compensation, optical-flow fusion, and background subtraction. SPGF incurs negligible online overhead because its scene prior is constructed only once for each fixed AOI, while online processing involves only prior lookup and candidate filtering. With a runtime of 3.161 s/frame, the current implementation does not yet meet the real-time processing requirement of WAMI systems operating at approximately 1–2 fps. Dense optical flow is currently executed on the CPU because the adopted OpenCV build does not provide CUDA optical-flow support, while the binary classifier remains the dominant computational bottleneck. Further acceleration could therefore be achieved through GPU-based optical-flow computation and more efficient candidate screening.

## 6. Discussion

The experimental results demonstrate that MDF-Det effectively addresses several key challenges in WAMI target detection. By integrating dense optical flow with grayscale differencing, MAFF enhances weak motion responses and improves the quality of foreground candidates. SA-TD further separates merged responses in densely populated regions through fine-grained heatmap decoding and step-threshold degradation, while SPGF introduces soft scene-level constraints to suppress false alarms caused by registration artifacts, vegetation motion, and other contextually implausible background responses. The ablation results confirm that the three components perform distinct yet complementary functions: MAFF shifts the detection balance toward higher Precision, SA-TD provides the largest gain in Recall and improves target separability, and SPGF further improves Precision through scene-level filtering.

Despite these advantages, MAFF remains dependent on the quality of inter-frame registration and optical-flow estimation. Strong parallax, abrupt illumination changes, or rapid camera motion may introduce residual misalignment or unreliable motion responses, thereby degrading candidate generation. MDF-Det also inherits a common limitation of candidate-driven cascaded pipelines. Since SA-TD and SPGF operate only on candidates retained by MAFF, weak targets completely missed during the initial candidate-generation stage cannot be recovered in subsequent refinement. Although the zero-shot transfer results indicate cross-scene transferability, SPGF is learned from trajectory-derived supervision within the training sequence and may therefore still exhibit some dependence on scene-specific spatial statistics.

A promising future direction is therefore to introduce a semantic feedback branch that complements the motion-based front end. By integrating motion confidence, foundation-model semantics, and soft road-network priors through uncertainty-aware gating, such a branch could reassess low-confidence regions and generate supplementary candidates in areas with weak motion responses but strong contextual evidence. This would provide a practical way to improve weak-target recovery while reducing the dependence of SPGF on scene-specific spatial statistics.

## 7. Conclusions

In this paper, we propose MDF-Det, a coarse-to-fine framework for tiny moving target detection in WAMI. By sequentially integrating motion–appearance fusion, spatial attention-guided target decoupling, and scene-prior filtering, MDF-Det effectively mitigates missed detections caused by weak motion responses, merged detections in dense scenes, and scene-induced false alarms. Experimental results on six evaluation AOIs of the WPAFB 2009 dataset demonstrate that MDF-Det achieves an average $F_{1}$ score of 0.878, corresponding to a relative improvement of 5.5% over the strongest baseline. These results demonstrate the effectiveness of MDF-Det across the six evaluated regions of the WPAFB benchmark.

Future work will focus on improving the cross-scene generalization of scene-prior modeling and reducing the dependence of the cascaded framework on the initial candidate-generation stage. We also plan to investigate semantic feedback mechanisms that integrate motion confidence, foundation-model semantics, and soft road-network priors, and to further validate MDF-Det on additional WAMI datasets and more diverse aerial imaging scenarios.

## Figures and Tables

**Table 1:** Principal implementation and training settings used for the reproduced baseline methods.

Method	Input Configuration	Training Configuration				Training Objective
		Optimizer	Learning Rate	Batch Size	Training	
ClusterNet [11]	Five consecutive frames; 128 × 128 × 5 patches	AdamW	$3\times 10^{-4}$	256	12 epochs	Focal binary cross-entropy; Dice loss
CNNs [15]	21 × 21 × 4 classification patches; 45 × 45 × 4 regression patches	SGD	$1\times 10^{-2}$ (cls.); $5\times 10^{-3}$ (reg.)	100	80 epochs (cls.); 100 epochs (reg.)	Cross-entropy for classification; MSE for heatmap regression
HMRN [17]	Current frame, previous frame; previous center heatmap	Adam	$1.25\times 10^{-4}$	16	75 epochs	CenterNet-style focal loss; center-masked displacement L1 loss
HM-Net [6]	Current frame, previous frame; previous heatmap	Adam	$3\times 10^{-4}$	6	40 epochs	Center focal loss; center-masked motion and precision L1 losses
CATLoss [34]	21 × 21 × 4 objectness patches; 45 × 45 × 4 localization patches	AdamW	$3\times 10^{-4}$	128 (obj.); 64 (loc.)	20 epochs	Binary focal loss for objectness; CATLoss for localization

**Table 2:** $F_{1}$ score comparison of different methods across six AOIs. The Average column reports the mean ± standard deviation over all six AOIs. The best results are highlighted in **bold**.

Method	AOIs						Average
	01	02	03	34	40	41	
ClusterNet [11]	0.805	0.901	0.779	0.869	0.792	0.775	0.820±0.052
CNNs [15]	0.691	0.799	0.558	0.774	0.681	0.726	0.705±0.085
HMRN [17]	0.769	0.821	0.800	0.878	0.746	0.751	0.794±0.050
HM-Net [6]	0.759	0.893	0.840	0.927	0.750	0.825	0.832±0.071
CATLoss [34]	0.743	0.660	0.748	0.765	0.729	0.743	0.731±0.037
**MDF-Det**	**0.848**	**0.914**	**0.888**	**0.941**	**0.835**	**0.843**	0.878±0.043

**Table 3:** Ablation study of the proposed MDF-Det framework on AOI03. The best detection results are highlighted in **bold**.

Modules			Metrics			
MAFF	SA-TD	SPGF	Recall	Precision	$F_{1}$	Time (s/Frame)
–	–	–	0.861	0.748	0.801	5.81
✓			0.807	0.800	0.803	3.54
	✓		**0.950**	0.753	0.840	5.66
		✓	0.861	0.771	0.813	5.58
✓	✓		0.933	0.838	0.883	**3.26**
	✓	✓	**0.950**	0.774	0.853	5.71
✓		✓	0.807	0.810	0.808	3.30
✓	✓	✓	0.933	**0.847**	**0.888**	3.32

**Table 4:** Internal ablation study of the SA-TD module on AOI03. The best detection results are highlighted in **bold**.

SA-TD Components		Metrics			
Spatial Attention	Threshold Degradation	Recall	Precision	$F_{1}$	Time (s/Frame)
–	–	**0.975**	0.604	0.746	3.15
–	✓	0.807	0.810	0.808	3.07
✓	–	0.958	0.817	0.882	3.16
✓	✓	0.933	**0.847**	**0.888**	3.32

**Table 5:** Validation-set performance under different combinations of the binary classification threshold $\tau_{\mathrm{bin}}$ and scene-prior confidence threshold $\tau_{\mathrm{prior}}$.

$\tau_{\mathrm{prior}}$	$\tau_{\mathrm{bin}}=0.5$			$\tau_{\mathrm{bin}}=0.7$			$\tau_{\mathrm{bin}}=0.9$		
	Recall	Precision	$F_{1}$	Recall	Precision	$F_{1}$	Recall	Precision	$F_{1}$
0.0	0.942	0.841	0.889	0.937	0.866	0.900	0.919	0.893	0.906
0.1	0.938	0.872	0.904	0.933	0.898	0.915	0.911	0.923	0.917
0.2	0.928	0.898	0.913	0.925	0.918	0.922	0.903	0.942	0.922
0.3	0.913	0.910	0.912	0.906	0.931	0.918	0.886	0.948	0.916
0.4	0.894	0.924	0.909	0.886	0.941	0.913	0.860	0.955	0.905

**Table 6:** Sensitivity of MDF-Det to different localization radii. The **bold** value denotes the radius adopted in the main experiments.

Radius (px)	Precision	Recall	$F_{1}$
5	0.941	0.862	0.900
10	0.948	0.874	0.909
15	0.950	0.879	0.913
**20**	**0.952**	**0.879**	**0.914**
25	0.954	0.883	0.917

**Table 7:** Computational complexity and runtime profile of MDF-Det on the validation set.

Module	Parameters	Model Size	FLOPs	Peak GPU Memory	Time	Runtime Contribution
	(M)	(MiB, FP32)	(G/Frame)	(MiB)	(s/Frame)	(%)
MAFF	0	–	–	–	0.864	27.32
Binary classifier	0.849	3.240	487.571	1176.0	1.621	51.28
SA-TD	8.337	31.803	2.498	84.2	0.676	21.40
SPGF	1.608	6.134	–	441.5	<0.001	0.01
Complete MDF-Det	10.794	41.177	490.069	1576.9	3.161	100.00

## Data Availability

The WPAFB 2009 and Greene 2007 datasets used in this study are publicly available from the U.S. Air Force Research Laboratory. The implementation of MDF-Det is available at https://github.com/likangqiushi20/MDF-Det (accessed on 8 September 2026).
